# Care for survivors when an emergency ends

**DOI:** 10.2471/BLT.25.294698

**Published:** 2026-01-01

**Authors:** Arham Kamil, Abdur Rafay Farooq

**Affiliations:** a Dow University of Health Sciences, Mission Rd, New Labour Colony Nanakwara, Karachi, Pakistan.

When a humanitarian crisis occurs, organizations, private individuals and the media mobilize. However, once the emergency passes, attention fades and survivors are often forgotten. Global health responses may save lives during crises but frequently fail to support survivors afterwards.

Survivorship entails living through the acute emergency but experiencing physical, psychological or social consequences in the aftermath. Recovery is the longer process of restoring or adapting health and functioning to make life liveable with dignity. These trajectories differ depending on the crisis, but in all cases, the emergency phase is only the beginning.

For example, following the 2013–2016 Ebola virus disease outbreak, over 17 000 individuals survived the viral infection but subsequently experienced chronic sequelae, including arthralgia, uveitis, memory loss and severe psychological trauma. Some survivors were stigmatized, deserted by their communities and offered limited organized care.^1^ In Mozambique, months after Cyclone Idai in 2019, maternal mortality increased as health infrastructure remained damaged and follow-up care was limited.^2^ Post-crisis reports highlighted these challenges, but long-term survivor programmes remained scarce. Similarly, after the 2022 floods in Pakistan, millions were displaced and faced heightened risks of malaria, diarrhoea and respiratory disease. When emergency shelters shut down, many families were left without reliable health care or secure housing.^3^^,^^4^

The 2023 Türkiye–Syrian Arab Republic earthquakes left thousands of survivors with amputations and spinal cord injuries. While national authorities and international humanitarian organizations prioritized trauma care, longer-term rehabilitation, prosthetic services and disability inclusion were often unfunded or implemented in fragmented efforts.^5^

The way the global health community structures crisis response partly contributes to this weakness. Donor finance and operational planning focus on preventing mortality, tracking case numbers and demonstrating rapid impact. Typically, no mandate and no budget exist for long-term health surveillance or continuity of care. Therefore, after the emergency phase, survivors often have no access to mental health services, physical rehabilitation or treatment for chronic conditions.^6^

However, considering this issue solely from an international perspective gives an incomplete picture. Continuity of post-disaster care is also a national responsibility. For instance, the 2023 Türkiye–Syrian Arab Republic earthquake revealed gaps in long-term rehabilitation and disability-inclusive recovery, even in Türkiye. These gaps suggest that the barrier is not simply a lack of donor funding, but one of national prioritization, planning and accountability.^7^ The Syrian Arab Republic faced compounded constraints, sanctions and security risks that complicated sustained assistance and limited what both national systems and external responders could realistically deliver over time.^8^ Even in settings with available resources, post-disaster care can be challenging. For example, after the 2015 Nepal earthquake, trained clinicians were reportedly unwilling to remain in remote affected areas, showing how health workforce distribution, incentives and governance can derail recovery even when emergency response is well funded.^9^

These shortcomings demonstrate that the problem is both a systems failure and an ethical issue. Health equity is breached when care is withheld from those who remain most in need after survival. Disaster survivors are frequently from disadvantaged groups such as rural residents, displaced persons, women and people with disabilities, and post-crisis abandonment deepens those disadvantages.

We call for the formal inclusion of survivorship into national plans for emergency preparedness, response and recovery, with support from international partners if needed. First, every emergency response should include a transparent transition plan into recovery care, with mechanisms to follow survivor outcomes over time. Second, dedicated funding for rehabilitation, post-traumatic mental health care and chronic disease management must be built into recovery planning. Third, national health systems must invest in the tools, workforce arrangements and partnerships required to maintain care long after the news fades.

Positive experiences show that these objectives are achievable. In Sierra Leone, government and partners created an Ebola survivor registry, extended free health care and launched the comprehensive package for Ebola survivors, which provides clinical and psychosocial services.^10^ After the 2008 Sichuan earthquake in China, a rehabilitation services programme delivered multiyear rehabilitation linked to improved functioning among people with disabling injuries.^11^ In Pakistan, army medical and engineering units have often supported disaster operations, illustrating how, when coordinated with civilian authorities, military assets can extend both immediate response and longer-term support.^12^ The Sphere project and the World Health Organization’s *Health emergency and disaster risk management framework* already emphasize continuity of care and national ownership. However, consistent, sufficiently funded implementation is still lacking.

Without a shift from short-term relief to sustained recovery, global health will remain reactive, and survivors will continue to face the long-term consequences of the crisis.
